# Introduction of a Protocol for Structured Follow-Up and Texting of Inadequate and Borderline-Positive Newborn Metabolic Screening Results

**DOI:** 10.3390/ijns8020030

**Published:** 2022-05-03

**Authors:** Natasha Heather, Lisa Morgan, Detlef Knoll, Keith Shore, Mark de Hora, Dianne Webster

**Affiliations:** 1 Newborn Metabolic Screening Programme, LabPlus, Auckland District Health Board, Auckland 1148, New Zealand; lisamo@adhb.govt.nz (L.M.); detlefk@adhb.govt.nz (D.K.); kshore@adhb.govt.nz (K.S.); mdehora@adhb.govt.nz (M.d.H.); diannew@adhb.govt.nz (D.W.); 2 Liggins Institute, University of Auckland, Auckland 1142, New Zealand

**Keywords:** newborn screening, neonatal screening, bloodspot sample, inadequate sample, borderline-positive test, repeat sample, SMS text message, screening follow-up, lost to follow-up

## Abstract

A national protocol for structured follow-up and texting of repeat newborn bloodspot screening (NBS) sample requests was introduced. Repeat samples are needed where the initial sample is inadequate or the result borderline-positive. This protocol aimed to improve the timeliness and completeness of receipt of repeat NBS samples. Under the structured protocol, all repeat sample requests were phoned or texted to the lead maternity carer (LMC), in addition to the standard written report issued. Weekly text reminders were sent until 4 weeks or the sample was received. National data were monitored following implementation of the protocol. The proportion of repeat samples received within 10 days of request improved after the introduction of the protocol, from 35.0% in 2013 to 81.4% in 2020 (*p* < 0.001). The proportion of requests lost to follow-up decreased, from 4.1% in 2013 to 1.3% in 2020 (*p* < 0.001). A structured NBS follow-up protocol that included SMS text messaging led to an earlier and more complete receipt of repeat samples. This is likely due to practitioners receiving the request more quickly, as well as the laboratory adopting a consistent approach to repeated reminders. SMS text messages are a useful adjunctive method for screening programmes to communicate with health care providers.

## 1. Introduction

Newborn bloodspot screening (NBS) aims to improve outcomes for affected infants through the early identification and treatment of disorders. Outcomes for many screened disorders are time critical, such that the timely completion of each step (sample collection, transport, testing, and result reporting), is crucial [1,2]. Most babies can be adequately screened from a single bloodspot sample; although, poor sample quality or out-of-range results can indicate the need for a further sample. If the initial sample is inadequate, commonly because of insufficient blood volume, sample recollection is required [3]. Repeat samples may also be required where the initial sample has a mild elevation in marker levels and is considered borderline-positive for a disorder. It is important that repeat samples are collected and processed in a timely way so that affected newborns can still be treated early if they have a disorder [4].

In New Zealand (NZ), routine NBS consists of a single heel-prick sample collected onto specialised collection paper within a recommended age of 48–72 h. A single screening laboratory in Auckland provides national coverage. Participation occurs with parental consent, with uptake > 99% of all births. Newborns are under the care of a lead maternity care (LMC, usually a midwife) until 6 weeks of age. LMCs are paid by the NZ government to care for the baby after birth, including the offer and provision of an NBS, or ensuring that this has occurred. The LMC is responsible for obtaining informed parental consent to participate in screening, sample collection, arranging transit to the screening laboratory, the communication of screen results, and any further recommended actions. The policy of the screening laboratory in NZ is that routine reporting and any additional contact occurs through the LMC, rather than directly with parents.

The national quality standard for repeat NBS samples is receipt by the screening laboratory within 10 calendar days of the request [5]. Where applicable, LMCs are asked to inform the laboratory if parents have declined the request, or if an alternative appropriate action has been performed. Of concern, less than 40% of requested repeat samples were received within the target timeframe in 2013 and 2014 [6,7], and up to 50 requests were lost to follow-up each year.

The time taken to receive a repeat sample is influenced by: the time for the report to be generated, mailed and received; the repeat sample to be collected (usually at the next scheduled LMC visit), mailed and received by the laboratory. Modifiable factors within the control of the screening laboratory include delays in paper reports reaching LMCs as well as ensuring that there is a consistent approach to communicating with LMCs and chasing up repeat sample requests.

The Clinical Laboratory Standards Institute Newborn Screening Follow-up Guideline (CLSI, NBS02) states that families and/or healthcare providers should be immediately notified of the need for a further sample [4]. They also recommend that screening programmes have protocols to confirm receipt and to chase up samples that have not arrived within the expected timeframe.

A structured protocol for the follow-up and communication of repeat NBS sample requests, utilising SMS text messages and regular reminders, was introduced. This protocol aimed to improve the timeliness and completeness of the receipt of repeat NBS samples requested by the laboratory.

## 2. Materials and Methods

### 2.1. Intervention

In May 2015, a new national protocol for the request for repeat NBS samples was introduced. This protocol added SMS text communication and a structured approach to the follow-up of samples not received within 7 days of a request from the laboratory.

NBS in New Zealand is overseen by the National Screening Unit (NSU), Ministry of Health. Permission to communicate patient information to LMCs via SMS text message was granted by the NZ privacy commissioner and NSU [8]. The process of texting LMCs and steps in the follow-up protocol were subsequently approved by the NSU Technical Working Group.

The screening laboratory developed a list of LMC phone numbers that were approved to receive SMS text messages. The approval process included confirmation of the phone number to which the LMC was willing to receive patient communication, and that the LMC would not share or allow others access to that phone.

Once approved for SMS text communication, LMCs received messages about inadequate samples via SMS text in addition to the routine paper report. The messages were standardised but able to be modified, and responses logged. Examples include:

*“Baby ABC NHI XYZ9999 newborn screening sample must be repeated because it has insufficient blood. Mark as repeat. Sample expected within 10 days. Phone xxxxxxxx if any queries. Thank-you”.*

*“Baby XYZ NHI XXX1234 newborn screening sample must be repeated because it is more than a month old. Mark as repeat. Sample expected within 10 days. You will receive a phonecall from the screening educator about this in the next day or so. Phone xxxxxxxx if any queries. Thank-you”.*

The process to communicate borderline-positive results was not changed. Following a borderline-positive result, LMCs continued to receive an explanatory phone call from the screening laboratory as well as a paper report.

In addition, the protocol introduced regular SMS text reminders (to approved LMCs) for samples that had not been received. Where samples were pending, reminders were sent at one, two, three and four weeks. The paper report was re-issued at three weeks, and if the request was still outstanding at four weeks, the reminder task was classified as lost to follow-up and closed by the laboratory.

Follow-up tasks were assigned by a specialised computer application developed for the NZ NBS laboratory in 2015. The application displayed tasks, including weekly reminders, on a daily dashboard. It logged SMS text messages sent to LMCs as well as any response received. Reminder tasks were performed by two trained laboratory staff members.

### 2.2. Monitoring

Repeat NBS sample requests before and after implementation of the structured follow-up protocol in May 2015 were monitored using national NBS data from 01/01/2013 to 31/12/2020. Samples requested from babies in neonatal intensive care units and/or where the laboratory was advised of subsequent neonatal death were excluded from the analysis.

Inadequate samples were defined as those requiring repeat collection due to a quality issue, for example, early collection (defined as <46 h of age until May 2020, from which point this was amended to <24 h), insufficient blood, or contamination. Borderline-positive results denoted samples where there was a minor elevation of screened metabolites and for which the disorder-specific screening algorithm included a request for a repeat sample.

The final outcomes were classified as either follow-up complete or incomplete (lost to follow-up). A follow-up complete outcome occurred where the repeat sample had been received, or the laboratory had received notification of parental decline to consent to the collection of a further sample, or that an alternative appropriate action had been performed. Such actions included specialist referral or further tests performed in a community laboratory (commonly thyroid function tests). Completed follow-up was further classified as having occurred within the target timeframe of ten calendar days from request, or outside of this. Loss to follow-up was assigned if follow-up remained incomplete after twenty-eight calendar days.

Following implementation of the structured follow-up protocol, the completion action leading to follow-up task closure was also monitored. Task closures were classified as occurring due to the requested sample being received, an alternative appropriate action performed, or notification of parental decline. This level of data was not consistently recorded prior to 2015.

Statistical analysis was largely descriptive, but chi-square tests were used to define statistically significant improvement, using a *p* value of <0.05.

## 3. Results

Between 2013 and 2020, a total of 469,723 babies were screened. The laboratory made 7494 repeat sample requests for inadequate samples or borderline-positive screen results (Table 1). A total of 6277 (83.8%) repeat sample requests were made for inadequate samples, with 1.0–1.8% of all initial samples classified as inadequate each year. The number of inadequate samples was greatest in 2014, following which the screening programme engaged in a series of education initiatives to improve sample quality.

Borderline-positive results accounted for 1184 (15.8%) of repeat sample requests, with the largest numbers occurring in 2013 and 2014 (Table 1). The number of requests decreased from 2015 onwards, alongside the introduction of second-tier tests for several amino acid breakdown disorders and for congenital adrenal hyperplasia. In addition to this, the screening programme stopped screening for three conditions with a high borderline-positive test rate (3-methylcrotonyl-CoA carboxylase deficiency 18 August 2015, tyrosinemia 18 June 2017, and carnitine uptake disorder 18 June 2017 [9].

Prior to 2015, compliance with the ten-day target timeframe for receipt (or other completed follow-up) of repeat sample requests was low, at 35.0% in 2013 and 38.2% in 2014 and increasing to 66.7% in 2015 (*p* < 0.001, Table 1). This early improvement was sustained over the following five years, such that 81.4% of requests met the timeframe in 2020. Of note, the laboratory also changed the transit method of NBS samples from routine mail to overnight courier delivery in 2016. However, the increase in the proportion of sample requests meeting timeframe between 2016 and 2017 was relatively minor (73.4% to 75.1%, *p* = 0.39).

The absolute number of repeat sample requests recorded as lost to follow-up was already low at baseline and prior to the structured follow-up intervention (<50 requests per year). However, further reduction was observed following the implementation of the structured follow-up protocol (Table 1). This trend towards fewer requests lost to follow-up became statistically significant in 2019 and 2020 (<10 requests per year, *p* < 0.001).

Following introduction of the protocol and a structured approach to recording outcomes, ≥95% of completed requests were closed due to receipt of the requested sample (Table 2). In addition to this, there were a small number of babies with borderline-positive results (2–6 cases each year) where an alternative appropriate action was known to have been performed. The proportion of requests that were declined by parents remained low and relatively consistent (132 instances, between 2% and 5%) between 2016 and 2020. Parental refusal almost always occurred following inadequate samples and was rare following a borderline-positive screen result.

## 4. Discussion

The aim of this quality improvement initiative was to reduce delays in the receipt of repeat NBS samples by improving communication with LMCs. We found that a structured follow-up protocol, which included the use of SMS text messages, led to the earlier receipt of samples and reduced the number of requests that were lost to follow-up. These are important outcomes, which can reduce delays in the identification and treatment of disorders detectable through screening.

Text messages are a common and convenient way for LMCs to communicate with their patients; however, this was the first time that the NBS laboratory had reported patient information this way. The risks of communicating health information via SMS text message were considered and a plan for mitigation of risk approved. SMS text message communication was additional to routine written reports, some of which are paper and sent by post and therefore take several days (or longer) to be received. Conversely, SMS text messages were received near instantaneously. The use of SMS text messages led to a dramatic improvement in the proportion of repeat samples received within 10 days of request. The timeliness of sample receipt continued to improve over the following years, potentially as the LMC workforce became increasingly familiar with receiving and acting on SMS texts from the screening laboratory.

A further key aspect of the follow-up protocol was a structured approach to the follow-up of sample requests. Follow-up tasks were assigned weekly through an NBS computer application. Although follow-up prior to this had occurred in an ad hoc manner, completion of follow-up was high (>95%) at baseline. Loss of follow-up can occur for a variety of reasons, and results in a baby that has not been reliably screened and is at risk of a poorer outcome through clinical presentation. Loss to follow-up decreased successively from 2017, with significant improvement observed in 2019 and 2020. This may have occurred as the intervention became well-established and the laboratory team increasingly familiar with the process.

In contrast, the parental refusal rate was not observed to change following introduction of the follow-up protocol. Participation in NBS in NZ is voluntary and occurs with parental consent. If a repeat sample is required, the laboratory communicates the request to the parents via the LMC. The laboratory supports the LMC with a verbal explanation and then respects the parental decision of whether to continue to participate in screening. This decision is largely outside of the control of the laboratory, and would not have been expected to have been impacted by the follow-up protocol. Parental refusal mostly occurred in response to inadequate samples and was rare following a borderline-positive result.

Second sample requests cause anxiety to families and lead to extra workload for both LMCs and the screening laboratory. In NZ, inadequate NBS samples are routinely tested so that disorder-probable results can be actioned in a timely way; however, a repeat sample is also requested. As noted, the number of borderline-positive test results has decreased due to increased use of second-tier testing, as well as the removal of three screened disorders with high borderline-positive test rates.

A key advantage of this quality improvement study is the availability of national prospectively collected observational data. Factors outside of the structured follow-up protocol, such as the switch from postal to courier sample transport, may also have contributed to the observed changes. The available data became more detailed and consistent following the introduction of the NBS computer application in mid-2015, which included a mechanism to track messages sent to and from LMCs, and to record sample receipt/parental decline/loss to follow-up in a consistent manner.

In summary, the structured follow-up protocol led to a more timely and complete receipt of requested repeat NBS samples. The use of SMS text messages improved the timeliness of communication between the NBS laboratory and LMCs and reduced delay in sample receipt, and the structured weekly approach to follow-up of outstanding requests decreased loss to follow-up.

The NZ antenatal screening programme is run through the same laboratory, and similar use of SMS texting to LMCs has been introduced to collect missing information and to report positive results, with the fail-safe of a response requested. In addition, further plans to develop the NBS computer application include a clinical follow-up module, which could similarly log communication with, and send information to, specialist paediatricians.

## Figures and Tables

**Table 1:** Repeat sample request outcomes 2013–2020.

	2013	2014	2015	2016	2017	2018	2019	2020
N babies screened	59,192	58,870	58,463	59,010	58,935	57,880	59,413	57,960
N inadequate samples (%screened)	711 (1.2%)	1034 (1.8%)	1020 (1.7%)	892 (1.5%)	730 (1.2%)	683 (1.2%)	574 (1.0%)	633 (1.1%)
N borderline-positive results	309	318	151	96	73	41	81	115
Total N 2nd sample requests	1020	1352	1171	988	836	714	655	748
N follow-up complete ≤ 10 days (%)	357 (35.0%)	517 (38.2%)	781 * (66.7%)	725 * (73.4%)	628 * (75.1%)	544 * (75.1%)	523 * (78.9%)	609 * (81.4%)
N follow-up complete > 10 days (%)	621 (60.9%)	800 (59.2%)	359 (30.7%)	236 (23.9%)	178 (21.3%)	156 (21.5%)	124 (18.9%)	129 (17.2%)
N follow-up complete ^†^ (%)	978 (95.9%)	1317 (97.4%)	1140 (97.4%)	961 (97.3%)	806 (96.4%)	700 (96.7%)	647 (98.8%)	738 (98.7%)
N lost to follow-up ^‡^ (%)	41 (4.1%)	35 (2.6%)	31 (2.6%)	27 (2.7%)	30 (3.6%)	24 (3.3%)	8 * (1.2%)	10 * (1.3%)

^†^ Follow-up complete = requested repeat samples received, declined or other appropriate follow-up notified to the screening laboratory. ^‡^ Lost to follow-up = allocated 28 days after the request for an initial sample if follow-up was not complete. * *p* < 0.001, as compared to 2013.

**Table 2:** Reason for task closure of completed repeat card requests, 2016–2020.

	2016	2017	2018	2019	2020
Repeat sample received (%)	926 (96.4%)	770 (95.5%)	665 (95.0%)	627 (96.9%)	712 (96.5%)
Parental refusal ^†^ (%)	29 (3.0%)	33 (4.1%)	33 (4.7%)	15 (2.3%)	22 (3.0%)
Alternative action ^‡^ (%)	6 (0.6%)	3 (0.4%)	2 (0.3%)	5 (0.8%)	4 (0.5%)
Total completed requests	961	806	700	647	738

^†^ Parental refusal to collect a requested sample that has been communicated to the screening laboratory. ^‡^ Alternative action includes a specialist referral and/or testing performed in a community laboratory (for example diagnostic serum thyroid function tests).

## Data Availability

The data presented in this study are available on request from the corresponding author, through application to the National Screening Unit, Ministry of Health.
